# Association of unsaturated fatty acid intake with risk of all-cause death in patients with osteoarthritis

**DOI:** 10.3389/fnut.2025.1454431

**Published:** 2025-04-07

**Authors:** Tianyang Li, Zhenzhou Tang, Sucheng Li, Weigang Jiang, Minhua Lu

**Affiliations:** Department of Orthopedics, The Fourth Affiliated Hospital to Soochow University (Suzhou Dushu Lake Hospital), Suzhou, China

**Keywords:** unsaturated fatty acid, death, osteoarthritis, NHANES, omega-6

## Abstract

**Aim:**

To investigate the relationship between unsaturated fatty acids (UFAs) intake and the risk of all-cause mortality in osteoarthritis (OA) patients.

**Methods:**

This cohort study included the data of 3,271 participants with OA using data from the National Health and Nutrition Examination Survey (NHANES). Univariate and multivariable weighted Cox regression models were applied to analyze the relationship between UFAs intake and the risk of mortality in patients with OA. Subgroup analysis was used in age, gender, cardiovascular disease (CVD), hypertension, and diabetes. Hazard ratio (HR), and 95% confidence interval (CI) were calculated.

**Results:**

The median follow-up time was 38.00 (69.00, 104.00) months, with 2,670 participants survived and 601 died. Monounsaturated fatty acids (MUFAs) ≥31.30 was associated with reduced risk of all-cause mortality in OA patients (HR = 0.48, 95% CI: 0.32–0.73). Lowered risk of all-cause mortality in OA patients was observed in patients with octadecenoic acid ≥29.14 (HR = 0.50, 95% CI: 0.34–0.72). Eicosenoic acid of 0.15–0.30 (HR = 0.70, 95% CI: 0.55–0.90) or eicosenoic acid ≥0.30 (HR = 0.62, 95% CI: 0.46–0.84) was related to decreased risk of all-cause mortality in OA patients. Polyunsaturated fatty acids (PUFAs) ≥20.33 was associated with reduced risk of all-cause mortality in OA patients (HR = 0.72, 95% CI: 0.54–0.96). Omega-3 fatty acid ≥1.98 was correlated with decreased risk of all-cause mortality in OA patients (HR = 0.60, 95% CI: 0.45–0.81). Decreased risk of all-cause mortality was found in people with alpha-linolenic acid (ALA) of 1.00–1.83 (HR = 0.75, 95% CI: 0.59–0.96) or ALA ≥1.83 (HR = 0.65, 95% CI: 0.46–0.92) in OA patients. Omega-6 fatty acid ≥18.04 (HR = 0.68, 95% CI: 0.51–0.92) or linoleic acid ≥17.89 (HR = 0.67, 95% CI: 0.50–0.90) were related to decreased risk of all-cause mortality in people with OA.

**Conclusion:**

Total MUFAs and PUFAs, octadecenoic acid, eicosenoic acid, omega-3 fatty acid, ALA, omega-6 fatty acid and linoleic acid were correlated with decreased risk of all-cause mortality in OA patients, which might suggest the importance of specific UFAs supplement in OA patients.

## Introduction

Osteoarthritis (OA) is one of the most important chronic diseases that leads to the degeneration and loss of cartilage within joints, resulting in a huge burden to patients and society (1). A study based on Global Burden of Disease (GBD) data revealed that OA had an annual global increase of 0.32% in age standardized incidence rate or approximately 9% increase over the 28-year period from 1990 to 2017 (2). Compared with the general population, patients with OA have a significantly increased risk of all-cause mortality (3). A number of observational studies have reported that people with OA are at increased risk of premature mortality compared to the general population (4, 5). The primary management of OA may necessitate sequential treatment (6), thus, to identify more reliable biomarkers associated with the prognosis of OA patients is of great value.

Previous studies highlighted the significance of joint-specific inflammation and oxidative stress in the progression and prognosis of OA (7–9), indicating that incorporating nutrients with anti-inflammatory properties into nutritional interventions may confer benefits. Unsaturated fatty acids (UFAs) are essential fatty acids for human body (10). Polyunsaturated fatty acids (PUFAs), especially omega-3 fatty acids, have been found to help improve pain, cartilage loss and joint function in OA patients due to their antioxidant and anti-inflammatory properties (11). Omega-3 fatty acids have been proposed as potential therapeutic agents for individuals with OA owing to their capacity to attenuate the systemic inflammatory response and foster an environment that inhibits cartilage degradation (11). In addition, studies have found that monounsaturated fatty acids (MUFAs) and PUFA might alleviate the progression of knee OA (12). However, whether UFAs played roles on the prognosis of patients with OA was still unclear.

This study aimed to investigate the relationship between UFAs intake and the risk of all-cause mortality in OA patients based on the data from National Health and Nutrition Examination Survey (NHANES). Subgroup analysis was performed in terms of age, gender, complicated with cardiovascular disease (CVD), diabetes, or hypertension.

## Methods

### Study design and population

In this cohort study, the records of 3,642 participants with OA were extracted from NHANES between 2007–2018. NHANES is an ongoing survey of non-institutionalized civilians residing in the United States, utilizing a complex sampling design that oversamples specific minority groups, income brackets, and age ranges (13). Data from NHANES are publicly available from https://wwwn.cdc.gov/nchs/nhanes/Default.aspx. Participants included should meet the following criteria: (1) ≥18 years old; (2) OA based on self-reported personal interview data. Participants were asked if they had ever been told by their doctor or another health professional that they had OA. The exclusion criteria of participants were: (1) missing survival data; (2) information of unsaturated fatty acid intake. Finally, 3,271 participants were analyzed.

### Main variables and outcome

Main variables were MUFAs including total MUFAs, hexadecenoic acid, octadecenoic acid (g), eicosenoic acid (g), and docosenoic acid (g), and PUFAs including total PUFAs, total omega-3 [eicosapentaenoic acid (EPA) + docosahexaenoic acid (DHA) + docosapentaenoic acid (DPA) + alpha-linolenic acid (ALA) + stearidonic acid (SDA)], EPA + DHA, DPA, ALA, SDA, total omega-6 (linoleic acid +arachidonic acid), linoleic acid, and arachidonic acid.

The food and nutrient intake of each participant in the NHANES database was recorded through a 24-h dietary recall interview. The 24-h dietary recall interview is comprised of two parts, the 24-h recall and a short set of post-dietary recall questions. Information collected from the 24-h recall interview will be coded and linked to a database of nutrient composition of foods. Calculations of total daily intakes of energy and 51 dietary components, including 19 individual fatty acids will be derived from these data. Following the dietary recall, the second part of the dietary interview will consist of a short questionnaire. The Food and Nutrient Database for Dietary Studies provided by the United States Department of Agriculture was utilized to calculate the consumption of UFAs (14). The intake of dietary supplements during the previous month, including dosage, frequency, and duration of consumption, was determined based on the questionnaire interview (15). The daily total intake of UFAs was determined by calculating the average of the two 24-h dietary intake records and dietary supplements intake.

All-cause mortality was the outcome in our study. The median follow-up time was 38.00 (69.00, 104.00) months.

### Potential covariates and definitions

Age (years), gender (males or females), race (White, Black or other races), education status (high school and below, or above high school), poverty to income ratio (PIR) (≤1.3, 1.3–1.85, or >1.85) (16, 17), marital status (married, widowed, divorced, separated, never married, or living with partner), smoking status (yes or no), drinking status (yes or no), physical activity [<450 metabolic equivalent (MET) × min/week or ≥450 MET × min/week], duration of arthritis, osteoporosis (yes or no), fracture (yes or no), hypertension (yes or no), diabetes (yes or no), dyslipidemia (yes or no), cardiovascular disease (CVD) (yes or no), chronic kidney disease (CKD) (yes or no), cancer (yes or no), BMI (<25 kg/m^2^, 25–30 kg/m^2^, or ≥30 kg/m^2^), energy, white blood cell count, uric acid, and non-steroidal anti-inflammatory agents (yes or no) were variables analyzed.

### Statistical analysis

All the data were analyzed in a weighted manner. The masked variance unit pseudo-stratum was sdmvstra, and the masked variance unit pseudo-primary sampling units was sdmvpsu. The confidence interval (CI) was applied for evaluating the reliability of an estimate. WTDRD1 was used as the dietary data weight. Continuous data were described as mean and standard error (S.E.), and the weighted *t*-test was used for comparison between groups. Enumeration data were described as the number and percentages of cases [*n* (%)], and *χ*^2^ test was used for comparison between groups. Univariate weighted Cox regression model was established to identify potential covariates associated the mortality of OA patients. Univariate and multivariable weighted Cox regression models were applied to analyze the relationship between UFAs intake and the risk of mortality in patients with OA. Missing values were manipulated via multiple imputation (Supplementary Table 1), and the results were compared between data with and without missing values imputation (Supplementary Table 2). Subgroup analysis was used in age, gender, CVD, hypertension, and diabetes. Hazard ratio (HR), and 95% confidence interval (CI) were calculated. Alpha was set as 0.05. Data analysis was generated using SAS 9.4.

## Results

### Comparisons of characteristics of participants survived or died

**Figure 1:**
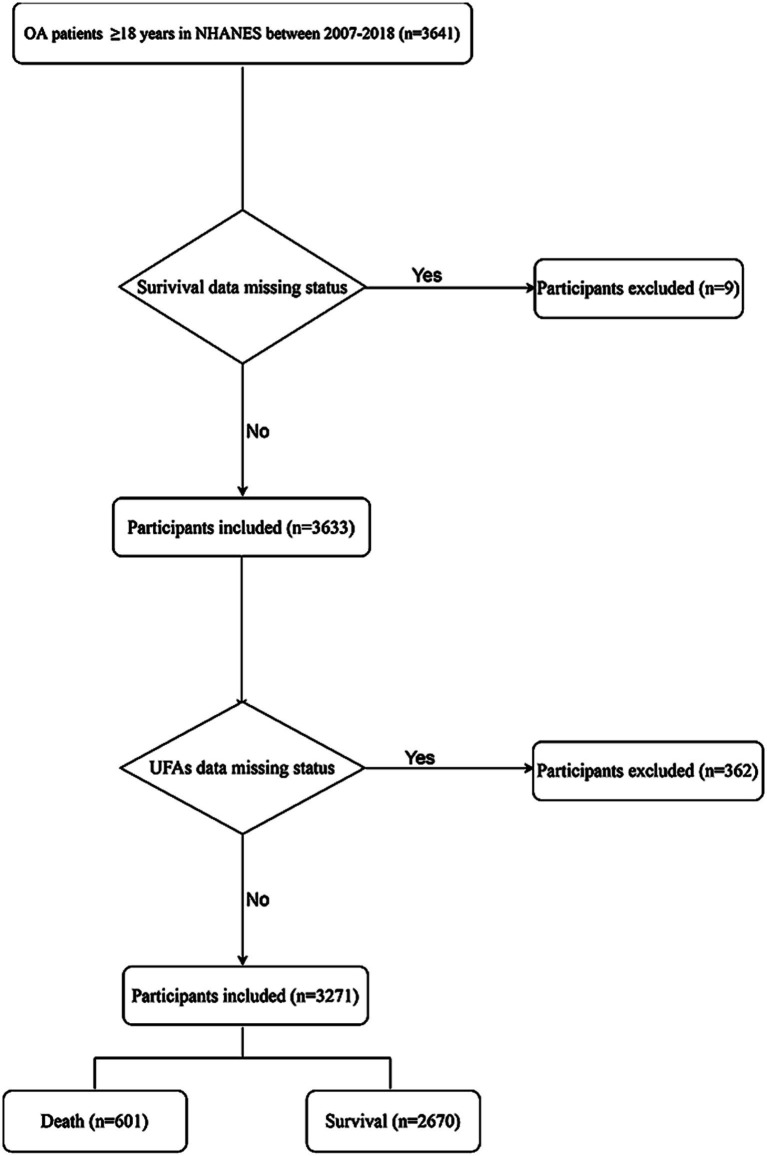
The screen process of the participants.

In total, the data of 3,642 participants were extracted from NHANES. Among them, participants without survival information were excluded (*n* = 9). Also, those without data on UFAs were not analyzed (*n* = 362). Finally, 3,271 subjects were involved in our study. There were 2,670 participants survived and 601 died. The screen process of the participants was depicted in Figure 1.

The mean MUFAs levels (27.88 g vs. 23.64 g, *p* < 0.001), hexadecenoic acid level (1.04 g vs. 0.91 g, *p* < 0.001), eicosenoic acid level (0.30 g vs. 0.22 g, *p* < 0.001), docosenoic acid level (0.04 g vs. 0.02 g, *p* = 0.005) in the survival group were higher than the death group, the mean omega-3 fatty acid level in the survival group was higher than the death group (1.88 g vs. 1.53 g, *p* < 0.001). Higher omega-6 fatty acid levels were observed in the survival group compared to the death group (16.29 g vs. 13.35 g, *p* < 0.001) (Table 1).

**Table 1:** Comparisons of characteristics of participants survived or died.

Variables	Total (*n* = 3,271)	All-cause mortality		Statistics	*p*
		Survival group (*n* = 2,670)	Death group (*n* = 601)		
Age (years), mean (S.E.)	61.76 (0.33)	60.33 (0.36)	70.59 (0.59)	*t* = −14.91	<0.001
Gender, *n* (%)				*χ*^2^ = 3.280	0.070
Male	1,185 (36.02)	927 (35.18)	258 (41.23)		
Female	2,086 (63.98)	1,743 (64.82)	343 (58.77)		
Race, *n* (%)				*χ*^2^ = 10.500	0.005
White	2,008 (81.72)	1,581 (81.07)	427 (85.80)		
Black	523 (6.80)	441 (6.92)	82 (6.08)		
Other	740 (11.48)	648 (12.02)	92 (8.13)		
Education status, *n* (%)				*χ*^2^ = 12.309	<0.001
High school and below	1,401 (36.10)	1,106 (34.83)	295 (43.99)		
Above high school	1,870 (63.90)	1,564 (65.17)	306 (56.01)		
PIR, *n* (%)				*χ*^2^ = 26.005	<0.001
≤1.3	876 (18.26)	713 (17.52)	163 (22.84)		
1.3–1.85	470 (11.83)	354 (10.94)	116 (17.37)		
1.85	1,925 (69.92)	1,603 (71.55)	322 (59.79)		
Marital status, *n* (%)				*χ*^2^ = 69.753	<0.001
Married	1,788 (60.55)	1,510 (62.61)	278 (47.79)		
Widowed	558 (13.81)	372 (11.33)	186 (29.23)		
Divorced	477 (13.08)	398 (13.01)	79 (13.51)		
Separated	87 (1.67)	72 (1.69)	15 (1.52)		
Never married	246 (7.46)	214 (7.77)	32 (5.55)		
Living with partner	115 (3.43)	104 (3.59)	11 (2.40)		
Smoking status, *n* (%)				*χ*^2^ = 6.211	0.013
No	1,565 (47.90)	1,310 (49.00)	255 (41.08)		
Yes	1,706 (52.10)	1,360 (51.00)	346 (58.92)		
Drinking status, *n* (%)				*χ*^2^ = 0.166	0.684
No	1,169 (30.26)	952 (30.12)	217 (31.17)		
Yes	2,102 (69.74)	1,718 (69.88)	384 (68.83)		
Physical activity (MET min/week), *n* (%)				*χ*^2^ = 79.230	<0.001
<450	1,563 (41.57)	1,178 (38.40)	385 (61.27)		
≥450	1,708 (58.43)	1,492 (61.60)	216 (38.73)		
Duration of arthritis, mean (S.E.)	12.59 (0.29)	12.06 (0.32)	15.84 (0.71)	*t* = −4.70	<0.001
Osteoporosis, *n* (%)				*χ*^2^ = 3.106	0.212
No	1,665 (50.17)	1,350 (50.31)	315 (49.34)		
Yes	10 (0.35)	4 (0.25)	6 (0.95)		
Unknown	1,596 (49.48)	1,316 (49.45)	280 (49.71)		
Fracture, *n* (%)				*χ*^2^ = 12.496	0.002
No	1,671 (47.85)	1,327 (47.19)	344 (51.89)		
Yes	406 (12.95)	313 (12.34)	93 (16.74)		
Unknown	1,194 (39.20)	1,030 (40.46)	164 (31.38)		
Hypertension, *n* (%)				*χ*^2^ = 44.892	<0.001
No	629 (23.13)	572 (25.18)	57 (10.38)		
Yes	2,642 (76.87)	2,098 (74.82)	544 (89.62)		
Diabetes, *n* (%)				*χ*^2^ = 22.270	<0.001
No	2,362 (77.85)	1,969 (79.36)	393 (68.43)		
Yes	909 (22.15)	701 (20.64)	208 (31.57)		
Dyslipidemia, *n* (%)				*χ*^2^ = 0.365	0.546
No	648 (19.36)	516 (19.19)	132 (20.42)		
Yes	2,623 (80.64)	2,154 (80.81)	469 (79.58)		
CVD, *n* (%)				*χ*^2^ = 33.514	<0.001
No	2,554 (81.67)	2,163 (83.85)	391 (68.13)		
Yes	717 (18.33)	507 (16.15)	210 (31.87)		
CKD, *n* (%)				*χ*^2^ = 116.423	<0.001
No	2,685 (85.83)	2,296 (88.59)	389 (68.69)		
Yes	375 (9.00)	213 (6.47)	162 (24.74)		
Unknown	211 (5.17)	161 (4.95)	50 (6.57)		
Cancer, *n* (%)				*χ*^2^ = 9.909	0.002
No	2,597 (78.01)	2,170 (79.15)	427 (70.88)		
Yes	674 (21.99)	500 (20.85)	174 (29.12)		
BMI, *n* (%)				*χ*^2^ = 7.445	0.024
<25	668 (20.78)	515 (20.00)	153 (25.62)		
25–30	1,001 (31.52)	795 (31.54)	206 (31.38)		
≥30	1,602 (47.70)	1,360 (48.46)	242 (42.99)		
Energy (kcal), mean (S.E.)	1947.05 (20.14)	1966.48 (21.98)	1826.38 (42.25)	*t* = 3.00	0.003
White blood cell count (1,000 cells/μL), mean (S.E.)	7.32 (0.06)	7.29 (0.06)	7.51 (0.14)	*t* = −1.52	0.133
Uric acid (μmol/L), mean (S.E.)	325.52 (1.82)	322.77 (1.96)	342.62 (5.05)	*t* = −3.69	<0.001
Non-steroidal anti-inflammatory agents, *n* (%)				*χ*^2^ = 8.617	0.003
No	2,847 (86.78)	2,292 (85.90)	555 (92.21)		
Yes	424 (13.22)	378 (14.10)	46 (7.79)		
MUFAs (g), mean (S.E.)	27.30 (0.41)	27.88 (0.46)	23.64 (0.65)	*t* = 5.29	<0.001
MUFAs (g), *n* (%)				*χ*^2^ = 23.263	<0.001
<19.09	1,155 (32.97)	909 (31.91)	246 (39.56)		
19.09–31.30	1,119 (33.96)	896 (33.15)	223 (39.01)		
≥31.30	997 (33.06)	865 (34.94)	132 (21.42)		
Hexadecenoic acid (g), mean (S.E.)	1.03 (0.02)	1.04 (0.02)	0.91 (0.03)	*t* = 3.44	<0.001
Hexadecenoic acid (g), *n* (%)				*χ*^2^ = 6.090	0.048
<0.63	1,115 (32.98)	887 (32.24)	228 (37.61)		
0.63–1.14	1,073 (33.96)	860 (33.73)	213 (35.37)		
≥1.14	1,083 (33.06)	923 (34.03)	160 (27.02)		
Octadecenoic acid (g), mean (S.E.)	25.37 (0.39)	25.91 (0.44)	22.03 (0.60)	*t* = 5.12	<0.001
Octadecenoic acid (g), *n* (%)				*χ*^2^ = 18.835	<0.001
<17.73	1,142 (32.96)	897 (31.87)	245 (39.74)		
17.73–29.14	1,125 (34.01)	909 (33.39)	216 (37.84)		
≥29.14	1,004 (33.03)	864 (34.73)	140 (22.43)		
Eicosenoic acid (g), mean (S.E.)	0.29 (0.01)	0.30 (0.01)	0.22 (0.01)	*t* = 5.92	<0.001
Eicosenoic acid (g), *n* (%)				*χ*^2^ = 31.011	<0.001
<0.15	1,136 (32.94)	863 (30.87)	273 (45.75)		
0.15–0.30	1,096 (34.05)	912 (34.59)	184 (30.68)		
≥0.30	1,039 (33.01)	895 (34.53)	144 (23.58)		
Docosenoic acid (g), mean (S.E.)	0.04 (0.00)	0.04 (0.00)	0.02 (0.00)	*t* = 2.89	0.005
Docosenoic acid (g), *n* (%)				*χ*^2^ = 20.429	<0.001
0	661 (16.99)	491 (15.70)	170 (25.01)		
0–0.013	1,339 (41.49)	1,105 (41.48)	234 (41.58)		
≥0.013	1,271 (41.51)	1,074 (42.82)	197 (33.41)		
PUFAs (g), mean (S.E.)	17.97 (0.28)	18.41 (0.32)	15.23 (0.46)	*t* = 5.76	<0.001
PUFAs (g), *n* (%)				*χ*^2^ = 23.919	<0.001
<11.64	1,154 (32.98)	889 (31.49)	265 (42.21)		
11.64–20.33	1,088 (33.99)	896 (34.06)	192 (33.59)		
≥20.33	1,029 (33.02)	885 (34.45)	144 (24.20)		
Omega-3 fatty acid (g), mean (S.E.)	1.83 (0.04)	1.88 (0.04)	1.53 (0.07)	*t* = 4.48	<0.001
Omega-3 fatty acid (g), *n* (%)				*χ*^2^ = 25.647	<0.001
<1.07	1,133 (33.00)	871 (31.51)	262 (42.22)		
1.07–1.98	1,106 (34.03)	897 (33.72)	209 (35.94)		
≥1.98	1,032 (32.97)	902 (34.77)	130 (21.84)		
EPA (g), mean (S.E.)	0.03 (0.00)	0.03 (0.00)	0.02 (0.00)	*t* = 2.66	0.009
EPA (g), *n* (%)				*χ*^2^ = 12.053	0.002
0	457 (13.35)	349 (13.15)	108 (14.58)		
0–0.007	1,417 (43.29)	1,153 (42.35)	264 (49.09)		
≥0.007	1,397 (43.36)	1,168 (44.50)	229 (36.33)		
DHA (g), mean (S.E.)	0.06 (0.00)	0.06 (0.00)	0.05 (0.01)	*t* = 2.35	0.021
DHA (g), *n* (%)				*χ*^2^ = 0.159	0.924
0	482 (14.20)	386 (14.15)	96 (14.55)		
0–0.018	1,306 (42.61)	1,084 (42.78)	222 (41.59)		
≥0.018	1,483 (43.18)	1,200 (43.07)	283 (43.86)		
EPA + DHA (g), mean (S.E.)	0.09 (0.01)	0.10 (0.01)	0.07 (0.01)	*t* = 2.55	0.012
EPA + DHA (g), *n* (%)				*χ*^2^ = 0.829	0.661
<0.01	1,039 (32.82)	831 (32.59)	208 (34.19)		
0.01–0.04	1,091 (34.02)	906 (33.87)	185 (34.93)		
≥0.04	1,141 (33.17)	933 (33.54)	208 (30.87)		
DPA (g), mean (S.E.)	0.02 (0.00)	0.02 (0.00)	0.02 (0.00)	*t* = 3.85	<0.001
DPA (g), *n* (%)				*χ*^2^ = 14.359	<0.001
<0.01	1,006 (29.54)	761 (28.05)	245 (38.80)		
0.01–0.02	1,191 (36.86)	1,003 (37.66)	188 (31.83)		
≥0.02	1,074 (33.61)	906 (34.29)	168 (29.37)		
ALA (g), mean (S.E.)	1.71 (0.04)	1.75 (0.04)	1.44 (0.07)	*t* = 4.11	<0.001
ALA (g), *n* (%)				*χ*^2^ = 18.311	<0.001
<1.00	1,147 (33.00)	877 (31.48)	270 (42.40)		
1.00–1.83	1,090 (33.88)	895 (33.86)	195 (33.97)		
≥1.83	1,034 (33.13)	898 (34.65)	136 (23.63)		
SDA (g), mean (S.E.)	0.01 (0.00)	0.01 (0.00)	0.01 (0.00)	*t* = 1.82	0.072
SDA (g), *n* (%)				*χ*^2^ = 6.204	0.045
0	1,723 (50.95)	1,361 (49.86)	362 (57.70)		
0–0.003	636 (21.17)	542 (21.66)	94 (18.10)		
≥0.003	912 (27.88)	767 (28.48)	145 (24.20)		
Omega-6 fatty acid (g), mean (S.E.)	15.89 (0.25)	16.29 (0.28)	13.35 (0.40)	*t* = 6.10	<0.001
Omega-6 fatty acid (g), *n* (%)				*χ*^2^ = 24.614	<0.001
<10.12	1,154 (32.86)	887 (31.34)	267 (42.29)		
10.12–18.04	1,089 (34.12)	894 (34.05)	195 (34.58)		
≥18.04	1,028 (33.02)	889 (34.61)	139 (23.12)		
Linoleic acid (g), mean (S.E.)	15.75 (0.25)	16.16 (0.28)	13.22 (0.40)	*t* = 6.12	<0.001
Linoleic acid (g), *n* (%)				*χ*^2^ = 25.510	<0.001
<10.04	1,155 (32.90)	887 (31.33)	268 (42.65)		
10.04–17.89	1,087 (34.08)	893 (34.06)	194 (34.23)		
≥17.89	1,029 (33.02)	890 (34.62)	139 (23.12)		
Arachidonic acid (g), mean (S.E.)	0.14 (0.00)	0.14 (0.00)	0.12 (0.01)	*t* = 1.84	0.069
Arachidonic acid (g), *n* (%)				*χ*^2^ = 5.113	0.078
<0.07	1,094 (32.93)	872 (32.16)	222 (37.71)		
0.07–0.15	1,080 (33.96)	881 (33.96)	199 (33.96)		
≥0.15	1,097 (33.11)	917 (33.88)	180 (28.32)		
Follow-up duration (month), mean (S.E.)	75.90 (1.83)	77.38 (1.98)	66.67 (3.30)	*t* = 3.08	0.003

PIR, poverty to income ratio; CVD, cardiovascular disease; CKD, chronic kidney disease; BMI, body mass index; MUFA, monounsaturated fatty acid; PUFA, polyunsaturated fatty acid; EPA, eicosapentaenoic acid; DHA, docosahexaenoic acid; DPA, docosapentaenoic acid; ALA, alpha-linolenic acid; SDA, stearidonic acid; S.E., standard error.

### Associations between UFAs with all-cause mortality in OA patients

As exhibited in Table 2, age, gender, education, PIR, marital status, smoking status, physical activity, duration of arthritis, osteoporosis, hypertension, diabetes, CVD, CKD, cancer, energy, WBC, uric acid, and non-steroidal anti-inflammatory agents were covariates associated with all-cause mortality in OA patients.

**Table 2:** Potential variables associated with the risk of all-cause mortality in OA patients.

Variables	HR (95% CI)	*p*
Age	1.08 (1.07–1.10)	<0.001
Gender		
Male	Ref	
Female	0.78 (0.61–0.99)	0.048
Race		
White	Ref	
Black	0.90 (0.71–1.14)	0.375
Other	0.77 (0.58–1.02)	0.073
Education status		
High school and below	Ref	
Above high school	0.73 (0.60–0.90)	0.003
PIR		
≤1.3	Ref	
1.3–1.85	1.10 (0.80–1.52)	0.536
>1.85	0.65 (0.52–0.82)	<0.001
Marital status		
Married	Ref	
Widowed	3.23 (2.47–4.24)	<0.001
Divorced	1.34 (0.94–1.91)	0.100
Separated	1.23 (0.63–2.38)	0.539
Never married	0.97 (0.55–1.71)	0.923
Living with partner	0.93 (0.41–2.08)	0.851
Smoking status		
No	Ref	
Yes	1.31 (1.05–1.63)	0.016
Drinking status		
No	Ref	
Yes	0.83 (0.68–1.02)	0.072
Physical activity (MET min/week)		
<450	Ref	
≥450	0.42 (0.34–0.51)	<0.001
Duration of arthritis	1.02 (1.01–1.03)	<0.001
Osteoporosis		
No	Ref	
Yes	8.39 (3.12–22.57)	<0.001
Unknown	1.50 (1.19–1.89)	<0.001
Fracture		
No	Ref	
Yes	1.31 (0.99–1.73)	0.055
Unknown	1.07 (0.80–1.43)	0.626
Hypertension		
No	Ref	
Yes	2.88 (2.13–3.88)	<0.001
Diabetes		
No	Ref	
Yes	1.94 (1.56–2.42)	<0.001
Dyslipidemia		
No	Ref	
Yes	0.99 (0.79–1.23)	0.908
CVD		
No	Ref	
Yes	2.52 (1.92–3.32)	<0.001
CKD		
No	Ref	
Yes	4.30 (3.32–5.58)	<0.001
Unknown	1.64 (1.09–2.47)	0.017
Cancer		
No	Ref	
Yes	1.67 (1.29–2.15)	<0.001
BMI		
<25	Ref	
25–30	0.82 (0.65–1.05)	0.120
≥30	0.85 (0.65–1.11)	0.233
Energy	0.84 (0.74–0.95)	0.005
White blood cell count	1.03 (1.01–1.06)	0.007
Uric acid	1.24 (1.10–1.40)	<0.001
Non-steroidal anti-inflammatory agents		
No	Ref	
Yes	0.58 (0.38–0.89)	0.013

OA, osteoarthritis; HR, hazard ratio; CI, confidence interval; PIR, poverty to income ratio; CVD, cardiovascular disease; CKD, chronic kidney disease; BMI, body mass index.

The results delineated that total MUFAs ≥31.30 (HR = 0.56, 95% CI: 0.41–0.76), hexadecenoic acid ≥1.14 (HR = 0.69, 95% CI: 0.52–0.93) might be associated with decreased risk of all-cause mortality in OA patients. Octadecenoic acid ≥29.14, eicosenoic acid of 0.15–0.30 (HR = 0.70, 95% CI: 0.55–0.89) or eicosenoic acid ≥0.30 might be correlated with reduced risk of all-cause mortality in OA patients. Docosenoic acid ≥0.013 (HR = 0.76, 95% CI: 0.58–0.99), PUFAs ≥20.33 (HR = 0.67, 95% CI: 0.53–0.84), omega-3 fatty acid ≥1.98 (HR = 0.61, 95% CI: 0.47–0.79), ALA ≥1.83 (HR = 0.66, 95% CI: 0.51–0.86), omega-6 fatty acid ≥18.04 (HR = 0.64, 95% CI: 0.50–0.81), or linoleic acid ≥17.89 (HR = 0.63, 95% CI: 0.49–0.80) might be related to lowered risk of all-cause mortality in OA patients (Table 3).

**Table 3:** Association between UFAs with all-cause mortality in OA patients.

Variables	Model 1		Model 2	
	HR (95% CI)	*p*	HR (95% CI)	*p*
MUFAs				
<19.09	Ref		Ref	
19.09–31.30	0.98 (0.77–1.25)	0.883	0.88 (0.68–1.14)	0.332
≥31.30	0.56 (0.41–0.76)	<0.001	0.48 (0.32–0.73)	<0.001
Hexadecenoic acid				
<0.63	Ref		Ref	
0.63–1.14	0.90 (0.70–1.15)	0.400	0.99 (0.76–1.29)	0.935
≥1.14	0.69 (0.52–0.93)	0.017	0.75 (0.53–1.07)	0.116
Octadecenoic acid				
<17.73	Ref		Ref	
17.73–29.14	0.96 (0.74–1.23)	0.735	0.87 (0.67–1.12)	0.271
≥29.14	0.57 (0.42–0.77)	<0.001	0.50 (0.34–0.72)	<0.001
Eicosenoic acid				
<0.15	Ref		Ref	
0.15–0.30	0.70 (0.55–0.89)	0.005	0.70 (0.55–0.90)	0.005
≥0.30	0.64 (0.49–0.83)	0.001	0.62 (0.46–0.84)	0.003
Docosenoic acid				
0	Ref		Ref	
0–0.013	0.93 (0.71–1.22)	0.585	0.89 (0.68–1.17)	0.407
≥0.013	0.76 (0.58–0.99)	0.048	0.77 (0.58–1.02)	0.072
PUFAs				
<11.64	Ref		Ref	
11.64–20.33	0.80 (0.63–1.01)	0.061	0.82 (0.63–1.07)	0.137
≥20.33	0.67 (0.53–0.84)	<0.001	0.72 (0.54–0.96)	0.025
Omega-3 fatty acid				
<1.07	Ref		Ref	
1.07–1.98	0.90 (0.69–1.17)	0.409	0.83 (0.65–1.07)	0.147
≥1.98	0.61 (0.47–0.79)	<0.001	0.60 (0.45–0.81)	<0.001
EPA + DHA				
<0.01	Ref		Ref	
0.01–0.04	1.07 (0.81–1.41)	0.638	1.20 (0.92–1.58)	0.180
≥0.04	0.91 (0.70–1.19)	0.489	0.99 (0.77–1.28)	0.973
DPA				
<0.01	Ref		Ref	
0.01–0.02	0.89 (0.67–1.20)	0.450	1.05 (0.80–1.39)	0.710
≥0.02	0.91 (0.70–1.18)	0.470	1.10 (0.87–1.40)	0.402
ALA				
<1.00	Ref		Ref	
1.00–1.83	0.84 (0.64–1.10)	0.205	0.75 (0.59–0.96)	0.024
≥1.83	0.66 (0.51–0.86)	0.002	0.65 (0.46–0.92)	0.017
SDA				
0	Ref		Ref	
0–0.003	0.89 (0.65–1.20)	0.435	1.05 (0.77–1.43)	0.735
≥0.003	0.78 (0.59–1.02)	0.071	0.90 (0.69–1.19)	0.466
Omega-6 fatty acid				
<10.12	Ref		Ref	
10.12–18.04	0.82 (0.64–1.06)	0.130	0.83 (0.63–1.10)	0.198
≥18.04	0.64 (0.50–0.81)	<0.001	0.68 (0.51–0.92)	0.012
Linoleic acid				
<10.04	Ref		Ref	
10.04–17.89	0.81 (0.63–1.04)	0.092	0.80 (0.61–1.06)	0.117
≥17.89	0.63 (0.49–0.80)	<0.001	0.67 (0.50–0.90)	0.008
Arachidonic acid				
<0.07	Ref		Ref	
0.07–0.15	0.94 (0.71–1.25)	0.686	1.01 (0.75–1.36)	0.949
≥0.15	0.91 (0.71–1.17)	0.447	1.02 (0.78–1.33)	0.884

HR, hazard ratio; CI, confidence interval; MUFA, monounsaturated fatty acid; PUFA, polyunsaturated fatty acid; EPA, eicosapentaenoic acid; DHA, docosahexaenoic acid; DPA, docosapentaenoic acid; ALA, alpha-linolenic acid; SDA, stearidonic acid. Model 1: crude model. Model 2 adjusting for age, gender, education, PIR, marital status, smoking status, physical activity, duration of arthritis, osteoporosis, hypertension, diabetes, CVD, CKD, cancer, energy, WBC uric acid, and non-steroidal anti-inflammatory agents (6, 16, 17).

After adjusting for confounding factors, MUFAs ≥31.30 was associated with reduced risk of all-cause mortality in OA patients (HR = 0.48, 95% CI: 0.32–0.73). Lowered risk of all-cause mortality in OA patients was observed in patients with octadecenoic acid ≥29.14 (HR = 0.50, 95% CI: 0.34–0.72). Eicosenoic acid of 0.15–0.30 (HR = 0.70, 95% CI: 0.55–0.90) or eicosenoic acid ≥0.30 (HR = 0.62, 95% CI: 0.46–0.84) was related to decreased risk of all-cause mortality in OA patients. PUFAs ≥20.33 was associated with reduced risk of all-cause mortality in OA patients (HR = 0.72, 95% CI: 0.54–0.96). Omega-3 fatty acid ≥1.98 was correlated with decreased risk of all-cause mortality in OA patients (HR = 0.60, 95% CI: 0.45–0.81). Decreased risk of all-cause mortality was found in people with ALA of 1.00–1.83 (HR = 0.75, 95% CI: 0.59–0.96) or ALA ≥1.83 (HR = 0.65, 95% CI: 0.46–0.92) in OA patients. Omega-6 fatty acid ≥18.04 (HR = 0.68, 95% CI: 0.51–0.92) or linoleic acid ≥17.89 (HR = 0.67, 95% CI: 0.50–0.90) were related to decreased risk of all-cause mortality in people with OA (Table 3).

### Subgroup analysis of associations between UFAs with all-cause mortality in OA patients

**Figure 2:**
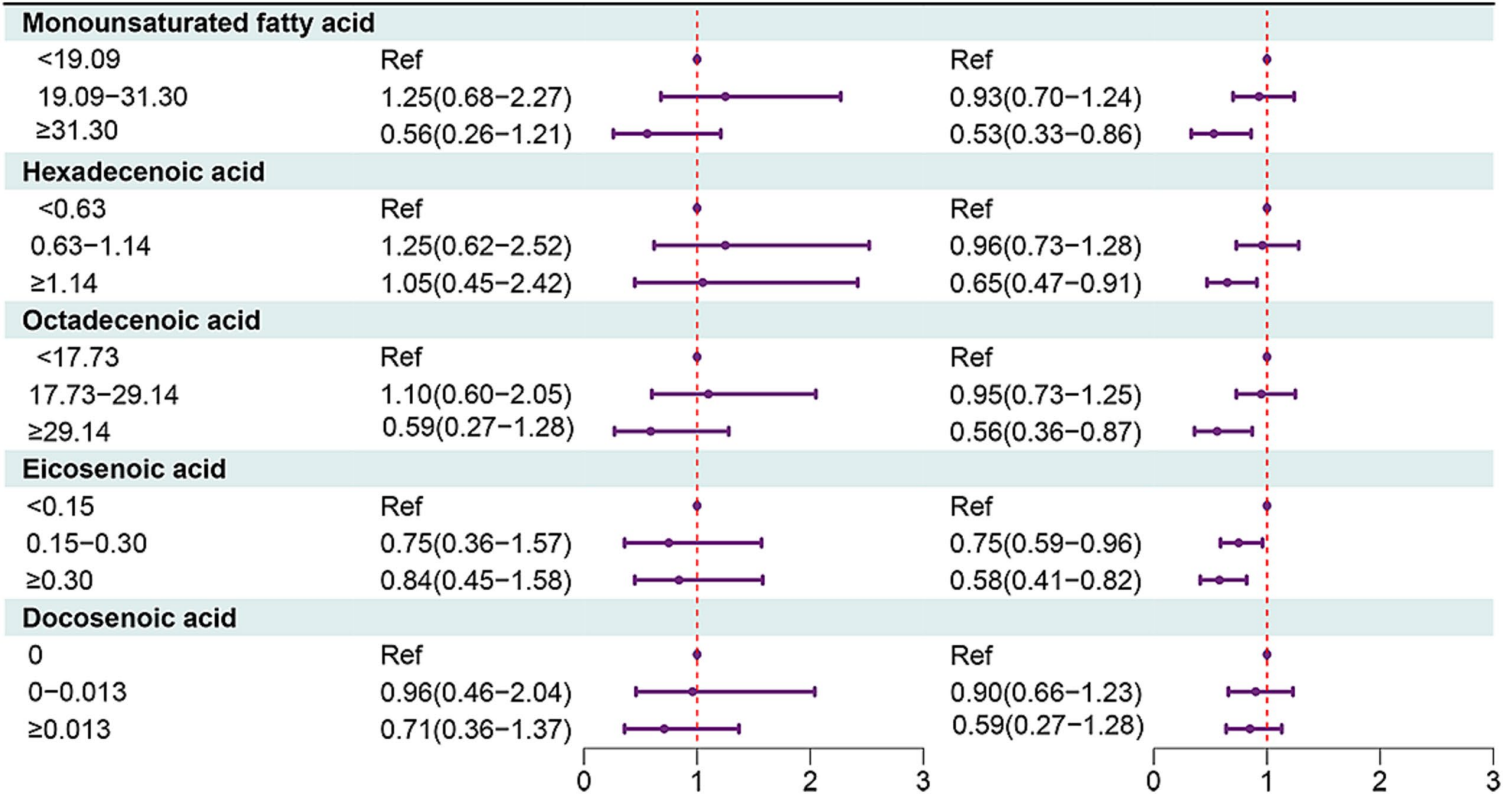
Forest plot showing the associations between MUFAs with all-cause mortality in OA patients in different age groups.

**Figure 3:**
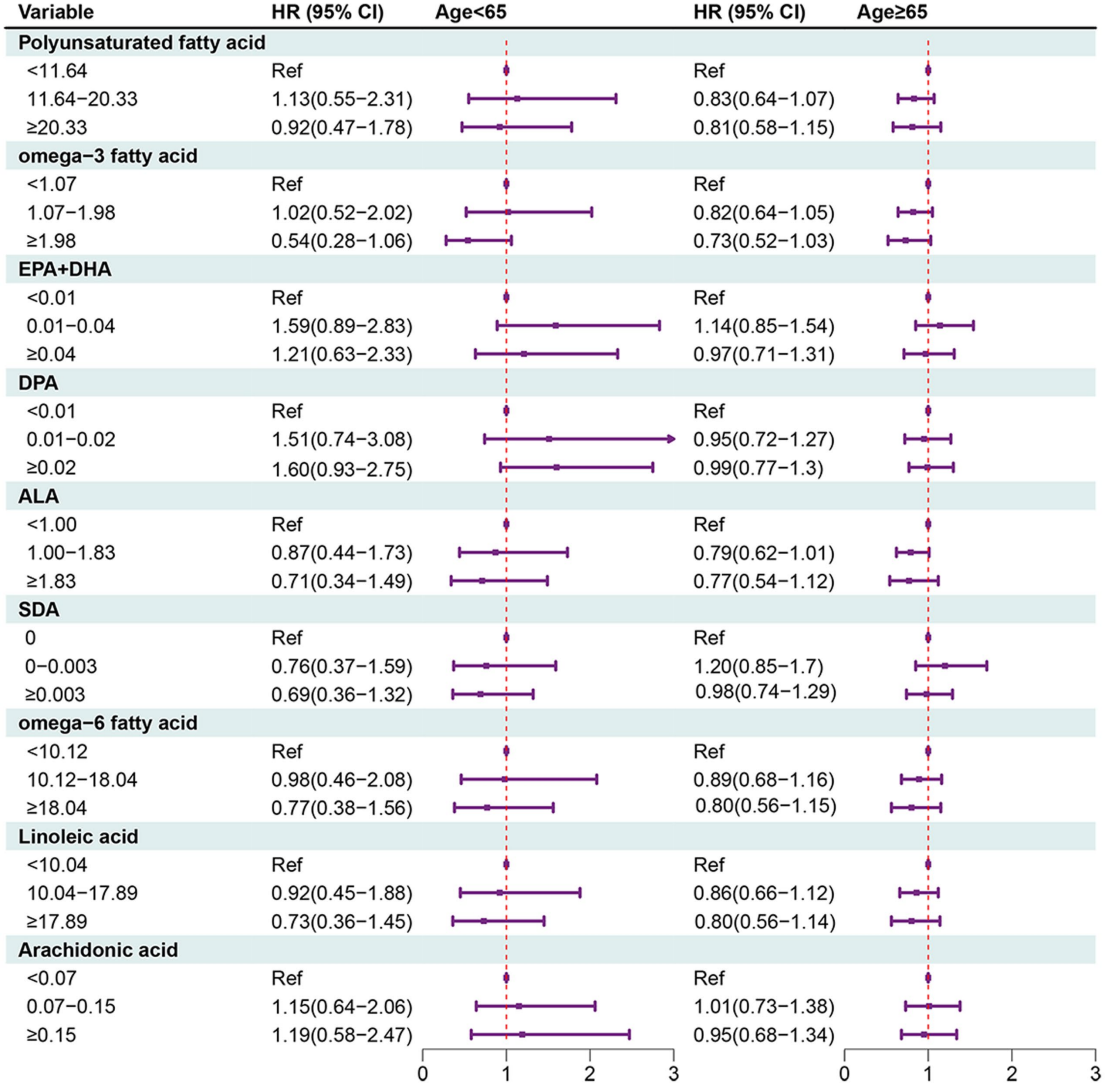
Forest plot showing the associations between PUFAs with all-cause mortality in OA patients in different age groups.

In people aged ≥65 years, MUFAs ≥31.30 (HR = 0.53, 95% CI: 0.33–0.86), hexadecenoic acid ≥1.14 (HR = 0.65, 95% CI: 0.47–0.91), octadecenoic acid ≥29.14 (HR = 0.56, 95% CI: 0.36 = 0.87), eicosenoic acid of 0.15–0.30 (HR = 0.75, 95% CI: 0.59–0.96) or eicosenoic acid ≥0.30 (HR = 0.58, 95% CI: 0.41–0.82) were associated with decreased risk of all-cause mortality in people with OA (Figures 2, 3).

**Figure 4:**
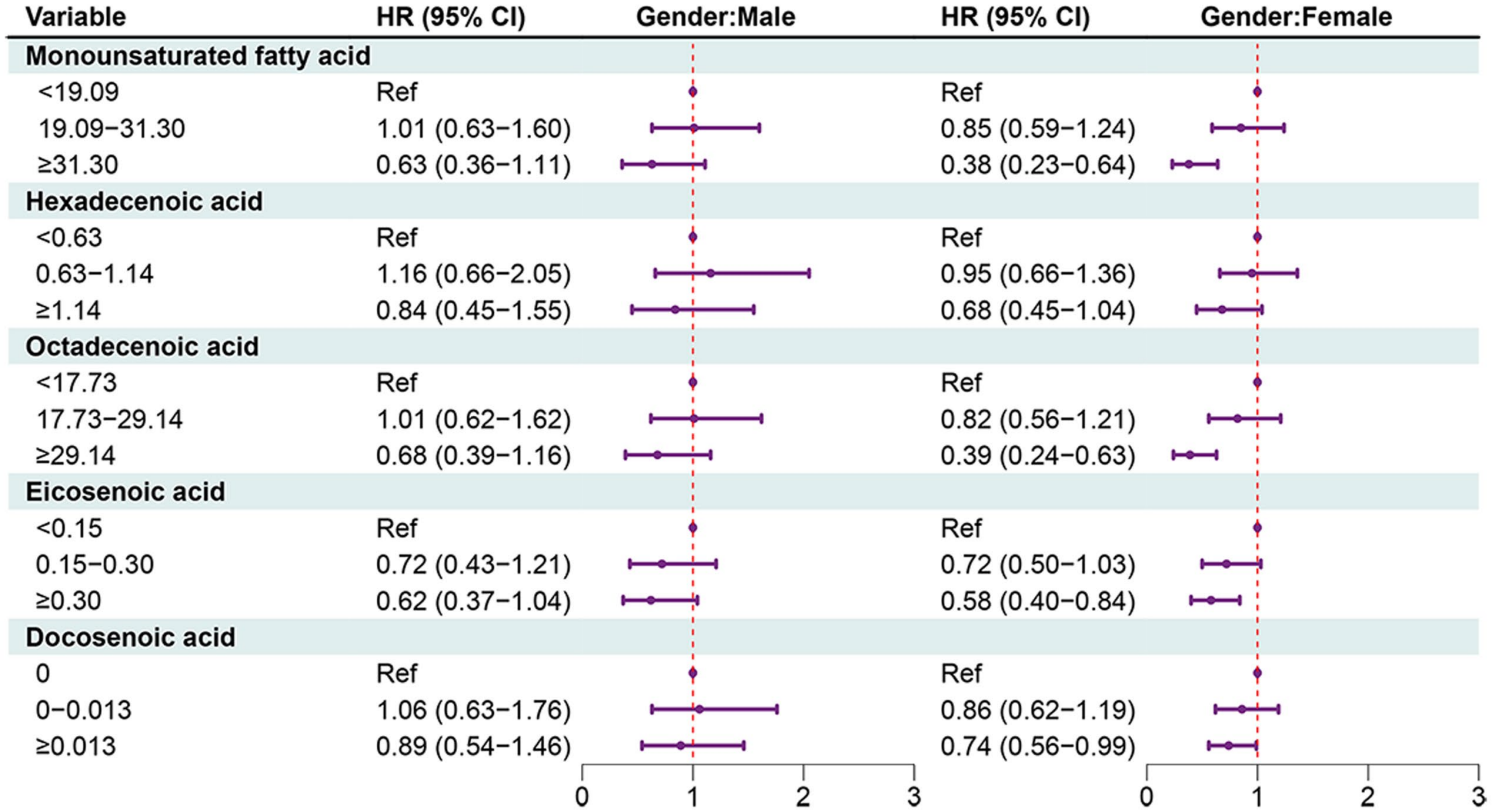
Forest plot showing the associations between MUFAs with all-cause mortality in OA patients in different gender groups.

**Figure 5:**
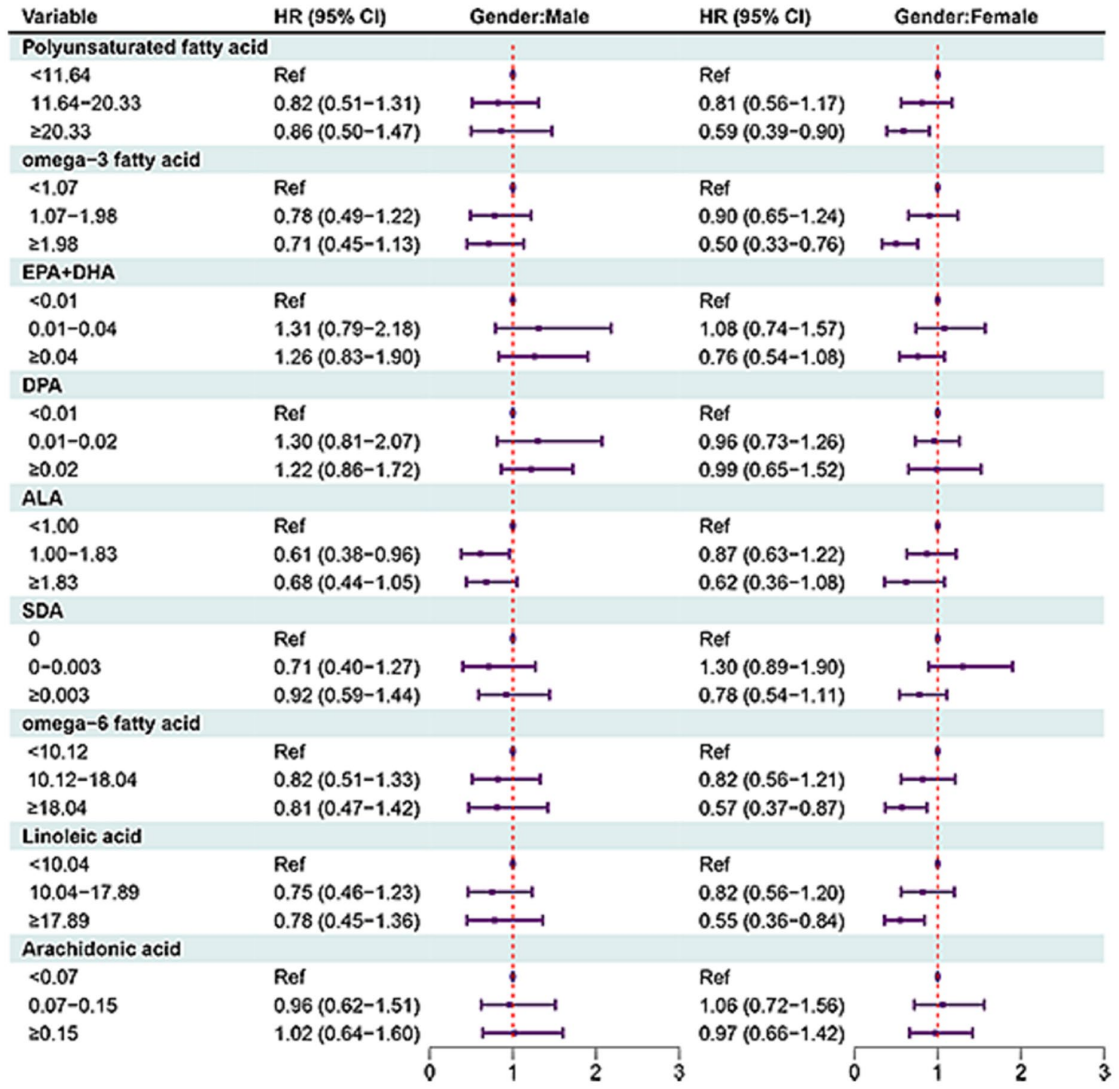
Forest plot showing the associations between PUFAs with all-cause mortality in OA patients in different gender groups.

In females, MUFAs ≥31.30 (HR = 0.38, 95% CI: 0.23–0.64), octadecenoic acid ≥29.14 (HR = 0.39, 95% CI: 0.24–0.63), eicosenoic acid ≥0.30 (HR = 0.58, 95% CI: 0.40–0.84), docosenoic acid ≥0.013 (HR = 0.74, 95% CI: 0.56–0.99). PUFAs ≥20.33 (HR = 0.59, 95% CI: 0.39–0.90), omega-3 fatty acid ≥1.98 (HR = 0.50, 95% CI: 0.33–0.76), omega-6 fatty acid ≥18.04 (HR = 0.57, 95% CI: 0.37–0.87), or linoleic acid ≥17.89 (HR = 0.55, 95% CI: 0.36–0.87) (Figures 4, 5).

## Discussion

The present study investigated the relationship between the levels of MUFAs and PUFAs intake and the risk of all-cause mortality in OA patients. The results depicted that total MUFAs ≥31.30, octadecenoic acid ≥29.14, and eicosenoic acid ≥0.15 as well as PUFAs ≥20.33, omega-3 fatty acid ≥1.98, ALA ≥1, omega-6 fatty acid ≥18.04 and linoleic acid ≥17.89 were related to decreased risk of all-cause mortality in OA patients. The finding showed directions for further studies to analyze the biologic and prognostic properties of MUFAs and PUFAs in OA patients.

In a previous study, a significant positive correlation of the consumption of saturated fatty acids, MUFAs, and PUFAs with bone mineral density was confirmed (18). MUFAs and PUFAs were reported to reduce radiographic progression of knee OA (12). Matsumoto et al. (19) revealed that MUFA, as a component of the Mediterranean diet score, was significantly lower in the rheumatoid arthritis, than in the control group and the ratio of consumed MUFA/SFA significantly differed within the rheumatoid arthritis group, which might suppress the disease activity in rheumatoid arthritis patients. Paunescu et al. (20) identified that MUFAs levels were positively associated with the stiffness index, and the status of MUFAs was associated with the strength of bone. Another cohort study identified that the HRs for total mortality when comparing the extreme quintiles of specific dietary fats were 0.81 (95% CI, 0.78–0.84) for PUFAs and 0.89 (95% CI, 0.84–0.94) for MUFAs. Replacing 5% of energy from saturated fats with an equivalent amount of energy from PUFAs and MUFAs was associated with estimated reductions in total mortality by 27% (HR, 0.73; 95% CI, 0.70–0.77) and 13% (HR, 0.87; 95% CI, 0.82–0.93), respectively (21). These findings might provide some support to the results in the current study. We found that total MUFAs ≥31.30, octadecenoic acid ≥29.14 and eicosenoic acid ≥0.15 were correlated with decreased risk of all-cause mortality of patients with OA, which suggested that that high MUFAs, octadecenoic acid, and and eicosenoic acid might help lower the mortality risk of OA patients.

Also, the relationship of PUFAs and bone health was illustrated in several studies. The potential therapeutic use of omega-3 fatty acids was postulated for individuals with OA, owing to their recognized anti-inflammatory properties, which could be beneficial in the context of OA by effectively moderating pro-inflammatory markers and mitigating cartilage loss (11). There was evidence indicated that the resolvin D1 (RvD1), a derivatives of omega-3 fatty acids, might participate in the pathogenesis of OA (22). The anti-inflammatory properties of omega-3 fatty acids are well recognized, which could be advantageous in the context of OA for modulating pro-inflammatory markers and mitigating cartilage loss (23, 24). A meta-analysis of randomized controlled trials revealed that the supplementation of omega-3 fatty acids has been proven effective in alleviating pain and enhancing joint function among patients with OA (25). The omega-3 fatty acids were reported to participate in certain mechanistic pathways involved in inflammation (26). The potential mechanisms underlying the beneficial effects of omega-3 fatty acids on OA are multifactorial. As previously reported, joint-specific inflammation plays a crucial role in the pathogenesis of OA (7), indicating that nutritional interventions incorporating anti-inflammatory nutrients may be beneficial. Omega-3 fatty acids are well-established for their potent anti-inflammatory properties (27). The alteration of cell membrane fatty acid composition, induced by the consumption of foods or supplements rich in omega-3 fatty acids, appears to play a crucial role in the anti-inflammatory effects attributed to omega-3 fatty acids (26). The omega-3 fatty acids were reported to participate in certain mechanistic pathways involved in inflammation (25). The alteration of cell membrane fatty acid composition, induced by the consumption of foods or supplements rich in omega-3 fatty acids, appears to play a crucial role in the anti-inflammatory effects attributed to omega-3 fatty acids (26). Omega-3 fatty acids exert anti-inflammatory effects by inhibiting inflammatory markers such as interleukin-1 beta (IL-1β) and inducible nitric oxide synthase (iNOS). Additionally, they suppress the expression of metalloproteinase 13, inhibit chondrocyte apoptosis, and restrain bone remodeling and angiogenesis within the osteochondral unit (28). The supplementation form of ALA significantly increased the content of bone-specific alkaline phosphatase, which was associated with bone health (29). A Mendelian randomization study indicated that there was causal relationship between PUFAs and OA susceptibility, and offered a novel insight that high omega-6 fatty acids might reduce the risk of knee OA and hip OA (30). These results were allied with the findings in our study, which delineated that PUFAs ≥20.33, omega-3 fatty acid ≥1.98, ALA ≥1, omega-6 fatty acid ≥18.04 and inoleic acid ≥17.89 were correlated with decreased risk of all-cause mortality in OA patients. High PUFAs including omega-3 fatty acid ALA omega-6 fatty acid and inoleic acid were potentially associated with the prognosis of OA patients. The causal associations still require validation in future studies.

Our study has a few notable advantages. This is the first study to examine the association between the intake of UFAs and the risk of all-cause mortality in patients with OA. The samples of this study was obtained from the NHANES database through multi-stage complex sampling, which represented the local population. The sample size was large and the follow-up time was long to ensure a sufficient number of outcome events. These results underscore the importance of maintaining the levels of UFAs in OA patients. Dietary recommendations advocating the role of UFAs and dietary guidelines should carefully consider the health effects of recommendations for UFAs in OA patients. The first limitation of this study was that this was a retrospective cohort study, and recall bias might exist. Second, due to the limitation of the NHANES database, to distinguish patients with OA at different sites was not possible. The association between UFAs intake and the risk of death in patients with OA at different sites needs to be further studied.

## Conclusion

The associations of MUFAs and PUFAs intake with the risk of all-cause mortality in OA patients were investigated in the present study. The results depicted that total MUFAs and PUFAs, octadecenoic acid, eicosenoic acid, omega-3 fatty acid, ALA, omega-6 fatty acid and linoleic acid were correlated with decreased risk of all-cause mortality in OA patients. The finding might suggest the importance of specific UFAs supplement in OA patients.

## Data Availability

Publicly available datasets were analyzed in this study. This data can be found here: NHANES database, https://wwwn.cdc.gov/nchs/nhanes/.

## References

[1] Salman LA Ahmed G Dakin SG Kendrick B Price A. Osteoarthritis: a narrative review of molecular approaches to disease management. Arthritis Res Ther. (2023) 25:27. doi: 10.1186/s13075-023-03006-w, PMID: 36800974 PMC9938549

[2] Jin Z Wang D Zhang H Liang J Feng X Zhao J. Incidence trend of five common musculoskeletal disorders from 1990 to 2017 at the global, regional and national level: results from the Global Burden of Disease Study 2017. Ann Rheum Dis. (2020) 79:1014–22. doi: 10.1136/annrheumdis-2020-217050, PMID: 32414807

[3] Constantino de Campos G Mundi R Whittington C Toutounji MJ Ngai W Sheehan B. Osteoarthritis, mobility-related comorbidities and mortality: an overview of meta-analyses. Ther Adv Musculoskelet Dis. (2020) 12:1759720x20981219. doi: 10.1177/1759720x20981219, PMID: 33488786 PMC7768583

[4] Nüesch E Dieppe P Reichenbach S Williams S Iff S Jüni P. All cause and disease specific mortality in patients with knee or hip osteoarthritis: population based cohort study. BMJ. (2011) 342:d1165. doi: 10.1136/bmj.d1165, PMID: 21385807 PMC3050438

[5] Liu R Kwok WY Vliet Vlieland TP Kroon HM Meulenbelt I Houwing-Duistermaat JJ. Mortality in osteoarthritis patients. Scand J Rheumatol. (2015) 44:70–3. doi: 10.3109/03009742.2014.922213, PMID: 25179456

[6] Healey EL Afolabi EK Lewis M Edwards JJ Jordan KP Finney A. Uptake of the NICE osteoarthritis guidelines in primary care: a survey of older adults with joint pain. BMC Musculoskelet Disord. (2018) 19:295. doi: 10.1186/s12891-018-2196-2, PMID: 30115048 PMC6097435

[7] Stannus O Jones G Cicuttini F Parameswaran V Quinn S Burgess J. Circulating levels of IL-6 and TNF-α are associated with knee radiographic osteoarthritis and knee cartilage loss in older adults. Osteoarthr Cartil. (2010) 18:1441–7. doi: 10.1016/j.joca.2010.08.016, PMID: 20816981

[8] Marchev AS Dimitrova PA Burns AJ Kostov RV Dinkova-Kostova AT Georgiev MI. Oxidative stress and chronic inflammation in osteoarthritis: can Nrf2 counteract these partners in crime? Ann N Y Acad Sci. (2017) 1401:114–35. doi: 10.1111/nyas.13407, PMID: 28662306

[9] Knights AJ Redding SJ Maerz T. Inflammation in osteoarthritis: the latest progress and ongoing challenges. Curr Opin Rheumatol. (2023) 35:128–34. doi: 10.1097/bor.0000000000000923, PMID: 36695054 PMC10821795

[10] Harwood JL. Polyunsaturated fatty acids: conversion to lipid mediators, roles in inflammatory diseases and dietary sources. Int J Mol Sci. (2023) 24:8838. doi: 10.3390/ijms24108838, PMID: 37240183 PMC10218335

[11] Cordingley DM Cornish SM. Omega-3 fatty acids for the management of osteoarthritis: a narrative review. Nutrients. (2022) 14:3362. doi: 10.3390/nu14163362, PMID: 36014868 PMC9413343

[12] Lu B Driban JB Xu C Lapane KL McAlindon TE Eaton CB. Dietary fat intake and radiographic progression of knee osteoarthritis: data from the osteoarthritis initiative. Arthritis Care Res. (2017) 69:368–75. doi: 10.1002/acr.22952, PMID: 27273934 PMC5140767

[13] Linakis MW Gustafson P Allen BC Bachand AM van Landingham C Keast DR. Is the cholesterol-perfluoroalkyl substance association confounded by dietary fiber intake?: a Bayesian analysis of NHANES data with adjustment for measurement error in fiber intake. Environ Health. (2022) 21:114. doi: 10.1186/s12940-022-00923-2, PMID: 36419083 PMC9682702

[14] Ahuja JK Moshfegh AJ Holden JM Harris E. USDA food and nutrient databases provide the infrastructure for food and nutrition research, policy, and practice. J Nutr. (2013) 143:241S–9S. doi: 10.3945/jn.112.170043, PMID: 23269654

[15] Kantor ED Rehm CD Du M White E Giovannucci EL. Trends in dietary supplement use among us adults from 1999–2012. JAMA. (2016) 316:1464–74. doi: 10.1001/jama.2016.14403, PMID: 27727382 PMC5540241

[16] Liu T Wang Y Meng T Ren Q Shi H Lin C. Association between cardiovascular health and all-cause mortality risk in patients with osteoarthritis. BMC Musculoskelet Disord. (2024) 25:641. doi: 10.1186/s12891-024-07729-y, PMID: 39143482 PMC11323624

[17] Lin Y Zeng G Sun Y. The joint effect of vitamin-D status and tobacco exposure on overweight and obesity in children. Br J Nutr. (2024) 132:1386–93. doi: 10.1017/S0007114524002071, PMID: 39501637 PMC11646676

[18] Fang ZB Wang GX Cai GZ Zhang PX Liu DL Chu SF. Association between fatty acids intake and bone mineral density in adults aged 20–59: NHANES 2011–2018. Front Nutr. (2023) 10:1033195. doi: 10.3389/fnut.2023.1033195, PMID: 37102128 PMC10123400

[19] Matsumoto Y Sugioka Y Tada M Okano T Mamoto K Inui K. Monounsaturated fatty acids might be key factors in the Mediterranean diet that suppress rheumatoid arthritis disease activity: the tomorrow study. Clin Nutr. (2018) 37:675–80. doi: 10.1016/j.clnu.2017.02.011, PMID: 28285975

[20] PaunescuACAyottePDewaillyEDodinS. Saturated and monounsaturated fatty acid status is associated with bone strength estimated by calcaneal ultrasonography in Inuit women from Nunavik (Canada): a cross-sectional study. J Nutr Health Aging. (2014) 18:663–71. doi: 10.1007/s12603-014-0498-0, PMID: 25226104

[21] Wang DD Li Y Chiuve SE Stampfer MJ Manson JE Rimm EB. Association of specific dietary fats with total and cause-specific mortality. JAMA Intern Med. (2016) 176:1134–45. doi: 10.1001/jamainternmed.2016.2417, PMID: 27379574 PMC5123772

[22] Benabdoune H Rondon EP Shi Q Fernandes J Ranger P Fahmi H. The role of resolvin D1 in the regulation of inflammatory and catabolic mediators in osteoarthritis. Inflamm Res. (2016) 65:635–45. doi: 10.1007/s00011-016-0946-x, PMID: 27056390

[23] Calder PC. Mechanisms of action of (n-3) fatty acids. J Nutr. (2012) 142:592s–9s. doi: 10.3945/jn.111.155259, PMID: 22279140

[24] Calder PC. Omega-3 fatty acids and inflammatory processes: from molecules to man. Biochem Soc Trans. (2017) 45:1105–15. doi: 10.1042/bst20160474, PMID: 28900017

[25] Deng W Yi Z Yin E Lu R You H Yuan X. Effect of omega-3 polyunsaturated fatty acids supplementation for patients with osteoarthritis: a meta-analysis. J Orthop Surg Res. (2023) 18:381. doi: 10.1186/s13018-023-03855-w, PMID: 37226250 PMC10210278

[26] Surette ME. The science behind dietary omega-3 fatty acids. CMAJ. (2008) 178:177–80. doi: 10.1503/cmaj.071356, PMID: 18195292 PMC2174995

[27] Richter CK Bowen KJ Mozaffarian D Kris-Etherton PM Skulas-Ray AC. Total long-chain n-3 fatty acid intake and food sources in the United States compared to recommended intakes: NHANES 2003–2008. Lipids. (2017) 52:917–27. doi: 10.1007/s11745-017-4297-3, PMID: 28956299

[28] Phitak T Boonmaleerat K Pothacharoen P Pruksakorn D Kongtawelert P. Leptin alone and in combination with interleukin-1-beta induced cartilage degradation potentially inhibited by EPA and DHA. Connect Tissue Res. (2018) 59:316–31. doi: 10.1080/03008207.2017.1385605, PMID: 28956662

[29] Dou Y Wang Y Chen Z Yu X Ma D. Effect of n-3 polyunsaturated fatty acid on bone health: a systematic review and meta-analysis of randomized controlled trials. Food Sci Nutr. (2022) 10:145–54. doi: 10.1002/fsn3.2655, PMID: 35035917 PMC8751426

[30] Li X Lu Z Qi Y Chen B Li B. The role of polyunsaturated fatty acids in osteoarthritis: insights from a Mendelian randomization study. Nutrients. (2023) 15:4787. doi: 10.3390/nu15224787, PMID: 38004181 PMC10674676

