# Supplementary material for: Association of unsaturated fatty acid intake with risk of all-cause death in patients with osteoarthritis
Source: Front Nutr. 2025 Apr 7;12:1454431. doi: 10.3389/fnut.2025.1454431 (PMC12009701; doi:10.3389/fnut.2025.1454431)
Supplement: Supplementary file 1 [file Table_1.docx]

Supplementary Table 1 The number and percentage of missing values of variables

| Variables | n (%) |
| --- | --- |
| Uric acid | 169 (5.17) |
| White blood cell count | 103 (3.15) |
| PIR | 258 (7.89) |
| Marital status | 1 (0.03) |
| Smoking status | 1 (0.03) |
| Drinking status | 180 (5.50) |
| Duration of arthritis | 129 (3.94) |
| Cancer | 3 (0.09) |
| BMI | 53 (1.62) |

PIR: poverty to income ratio; BMI: body mass index

Supplementary Table 2 Sensitivity analysis of missing values manipulation

| Variables | After interpolation (n=3271) | Before interpolation (n=3271) | Statistics | *P* |
| --- | --- | --- | --- | --- |
| BMI, n (%) |  |  | χ^2^=5.014 | 0.082 |
| <25 | 668 (20.78) | 662 (20.91) |  |  |
| 25-30 | 1001 (31.52) | 982 (31.58) |  |  |
| ≥30 | 1602 (47.70) | 1574 (47.51) |  |  |
| Uric acid (umol/L), Mean (S.E) | 325.52 (1.82) | 325.29 (1.98) | t=0.51 | 0.615 |
| White blood cell count (1000 cells/uL), Mean (S.E) | 7.32 (0.06) | 7.33 (0.06) | t=-1.95 | 0.055 |
| PIR, n (%) |  |  | χ^2^=4.770 | 0.092 |
| ≤1.3 | 876 (18.26) | 812 (17.79) |  |  |
| 1.3-1.85 | 470 (11.83) | 437 (11.60) |  |  |
| ≥1.85 | 1925 (69.92) | 1764 (70.61) |  |  |
| Marital status, n (%) |  |  | χ^2^=5.060 | 0.409 |
| Married | 1788 (60.55) | 1787 (60.54) |  |  |
| Widowed | 558 (13.81) | 558 (13.82) |  |  |
| Divorced | 477 (13.08) | 477 (13.09) |  |  |
| Separated | 87 (1.67) | 87 (1.67) |  |  |
| Never married | 246 (7.46) | 246 (7.46) |  |  |
| Living with partner | 115 (3.43) | 115 (3.43) |  |  |
| Drinking status, n (%) |  |  | χ^2^=0.443 | 0.506 |
| No | 1169 (30.26) | 1109 (30.40) |  |  |
| Yes | 2102 (69.74) | 1982 (69.60) |  |  |
| Smoking status, n (%) |  |  | χ^2^=0.995 | 0.319 |
| No | 1565 (47.90) | 1564 (47.84) |  |  |
| Yes | 1706 (52.10) | 1706 (52.16) |  |  |
| Duration of arthritis, Mean (S.E) | 12.59 (0.29) | 12.61 (0.30) | t=-0.81 | 0.421 |
| Cancer, n (%) |  |  | χ^2^=1.655 | 0.198 |
| No | 2597 (78.01) | 2594 (77.99) |  |  |
| Yes | 674 (21.99) | 674 (22.01) |  |  |

PIR: poverty to income ratio; BMI: body mass index
